# Clinical Practice Guideline for *Tripterygium* Glycosides/*Tripterygium wilfordii* Tablets in the Treatment of Rheumatoid Arthritis

**DOI:** 10.3389/fphar.2020.608703

**Published:** 2021-01-14

**Authors:** Na Lin, Yan-Qiong Zhang, Quan Jiang, Wei Liu, Jian Liu, Qing-Chun Huang, Kuan-Yu Wu, Sheng-Hao Tu, Zu-Shan Zhou, Wei-Heng Chen, Xiao-Xia Li, Ying Ding, Yong-Fei Fang, Jian-Ping Liu, Zhen-Bin Li, Dong-Yi He, Yao-Long Chen, Yu-Qian Lou, Qing-Wen Tao, Qing-Wen Wang, Ying-Hui Jin, Xing Liao, Tai-Xian Li, Xiao-Yue Wang

**Affiliations:** ^1^ Institute of Chinese Materia Medica, China Academy of Chinese Medical Sciences, Beijing, China; ^2^ Guang’anmen Hospital, China Academy of Chinese Medical Sciences, Beijing, China; ^3^ First Teaching Hospital of Tianjin University of Traditional Chinese Medicine, Tianjin, China; ^4^ First Affiliated Hospital of Anhui University of Traditional Chinese Medicine, Hefei, China; ^5^ Guangdong Provincial Hospital of Traditional Chinese Medicine, Guangzhou, China; ^6^ Second People’s Hospital, Fujian University of Traditional Chinese Medicine, Fuzhou, China; ^7^ Tongji Hospital Affiliated to Tongji Medical School, Huazhong University of Science and Technology, Wuhan, China; ^8^ Department of Honghu, Hubei Province Hospital of Traditional Chinese Medicine, Honghu, China; ^9^ Third Affiliated Hospital of Beijing University of Chinese Medicine, Beijing, China; ^10^ Xuanwu Hospital, Capital Medical University, Beijing, China; ^11^ First Affiliated Hospital of the Henan University of Traditional Chinese Medicine, Zhengzhou, China; ^12^ Southwest Hospital, Army Medical University, Chongqing, China; ^13^ Centre for Evidence-Based Chinese Medicine, Beijing University of Chinese Medicine, Beijing, China; ^14^ Bethune International Peace Hospital, People’s Liberation Army, Shijiazhuang, China; ^15^ Shanghai Guanghua Hospital of Integrated Traditional and Western Medicine, Shanghai, China; ^16^ Evidence-Based Medicine Center, School of Basic Medical Sciences, Lanzhou University, Lanzhou, China; ^17^ Henan Rheumatism Hospital, Zhengzhou, China; ^18^ China-Japan Friendship Hospital, Beijing, China; ^19^ Shenzhen Hospital, Peking University, Shenzhen, China; ^20^ Center for Evidence-Based and Translational Medicine, Zhongnan Hospital of Wuhan University, Wuhan, China; ^21^ Institute of Basic Research in Clinical Medicine, China Academy of Chinese Medical Sciences, Beijing, China

**Keywords:** *Tripterygium* glycoside tablet, *Tripterygium wilfordii* tablets, rheumatoid arthritis, clinical practice guideline, rational drug use

## Abstract

*Tripterygium* *wilfordii* Hook F (*Tw*HF) is one of the most commonly used and effective traditional Chinese herbal medicines against rheumatoid arthritis (RA). Both Tripterygium Glycoside Tablets (TGT) and *Tripterygium wilfordii* Tablets (TWT) are the representative *Tw*HF-based agents enrolled into the 2019 edition of Medicine Catalog for National Basic Medical Insurance, Injury Insurance, and Maternity Insurance. However, individual differences in TGT/TWT response across patients usually exist in the process of treating RA, implying that the clinical application of the two agents may not be standardized leading to the ineffective treatment and the risk of side effects. Growing evidence show that the bioactive constituents of *Tw*HF may often have toxicity, the package insert of TGT and TWT may not be described in detail, and the therapeutic windows of the two agents are narrow. Thus, it is an urgent task to develop a standardized clinical practice guideline for TGT and TWT in the treatment of RA. In the current study, a group of clinical experts of traditional Chinese medicine and Western medicine in the research field of rheumatism diseases, pharmacists, and methodologists of evidence-based medicine were invited to select the clinical questions, to determine the levels of the evidence and the strength of the recommendations, and to develop the recommendations and good practice points. The guideline is formed based on the combination of clinical research evidence and expert experience (evidence-based, consensus, supplemented by experience). The clinical problems which are supported by clinical evidence may form recommendations, and the clinical problems without clinical evidence may form experts’ suggestions. Both recommendations and experts' suggestions in this guideline summarized the clinical indications, usage, dosage, combined medication, and safety of TGT and TWT against RA systematically and comprehensively, which may offer a professional guidance in the context of the clinical application of the two *Tw*HF-based agents.

## Introduction

Rheumatoid arthritis (RA) is a common chronic autoimmune disorder characterized by synovitis, cartilage damage, and bone erosion (Chimenti et al., 2015). The morbidity of RA is 0.19–0.41% in China. During the progression of the disease, the damage of multiple joints may lead to deformity and disability, which severely influences the patients’ quality of life and eventually leads to heavy social and economic burden (Wang et al., 2015). Although various conventional disease-modifying antirheumatic drugs (DMARDs) have been extensively used for the treatment of RA worldwide, the incidence of adverse reactions due to the application of DMARDs has been over 40% (Pavy et al., 2006; Li et al., 2016; Smolen et al., 2017).

*Tripterygium wilfordii* Hook F (*Tw*HF) is one of the most commonly used medicines and has been widely used as an effective traditional Chinese herbal medicine against RA in China for centuries (Zhang et al., 2010). Both Tripterygium Glycoside Tablets (TGT) and *Tripterygium wilfordii* Tablets (TWT) are the representative *Tw*HF-based agents enrolled into the 2019 edition of Medicine Catalog for National Basic Medical Insurance, Injury Insurance, and Maternity Insurance. Chemically, TGT mainly contains wilforlide A, triptolide, triptonide, wilforine, triptophenolide, tripterine, *etc.* (Wang Y.D. et al., 2019). Similarly, the constituents in TWT include triptolide, tripterine, wilforlide A, wtlfordine, triptophenolide, *etc.* (Gu et al., 2015). Among them, wilforlide A, triptolide, and tripterine have been demonstrated to exert good anti-inflammatory and immune inhibitory effects (Zhu et al., 2009; Xue et al., 2010; Venkatesha et al., 2012), which make both TGT and TWT be commonly used against RA. However, individual differences in TGT/TWT response across patients usually exist in the process of treating RA, implying that the clinical application of the two agents may not be standardized leading to the ineffective treatment and the risk of side effects. Growing evidence shows that the bioactive constituents of *Tw*HF may often have toxicity, the package insert of TGT and TWT may not be described in detail, and the therapeutic windows of the two agents are narrow. Thus, it is an urgent task to develop a standardized clinical practice guideline for TGT and TWT in the treatment of RA.

With the approval and support of the Standardization Office of the Chinese Association of Chinese Medicine, in the current study, we invited a group of clinical experts of traditional Chinese medicine and Western medicine in the research field of rheumatism diseases, pharmacists, and methodologists of evidence-based medicine to select the clinical questions, to determine the levels of the evidence and the strength of the recommendations, and to develop the recommendations and good practice points (GPPs). The guideline is formed based on the combination of clinical evidence and experts’ experience (evidence-based, consensus, supplemented by experience). The clinical problems which are supported by clinical evidence may form recommendations, and the clinical problems without clinical evidence may form experts' suggestions. Both recommendations and experts’ suggestions in this guideline summarized the clinical indications, usage, dosage, combined medication, and safety of TGT and TWT against RA systematically and comprehensively, which may offer a professional guidance in the context of the clinical application of the two *Tw*HF-based agents (Lin et al., 2020a; Lin et al., 2020b).

## Methods

**FIGURE 1:**
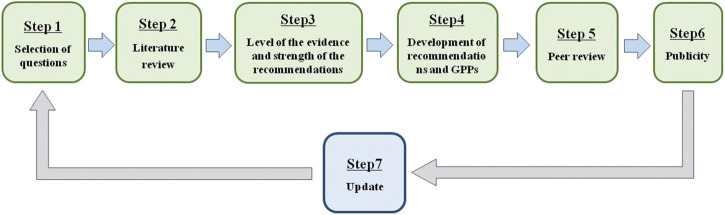
The procedure used to develop the clinical practice guideline.

The “Clinical practice guideline for Tripterygium Glycosides/*Tripterygium wilfordii* Tablets in the treatment of rheumatoid arthritis” was approved on June 2020 by the Standardization Office of the Chinese Association of Chinese Medicine. To develop an evidence-based and expert-approved Chinese patent medicine treatment guideline for RA, a multidisciplinary guideline development group was established. This guideline was developed according to the Manual for the clinical experts consensus of Chinese patent medicine (Fang et al., 2018). GPP (Liao and Xie, 2008) and the grading of recommendation assessment, development, and evaluation (GRADE) system (Atkins et al., 2004) were used to rate the quality of evidence and the strength of recommendations. Our clinical practice guideline was developed following a six-step plan (Figure 1).

### Selection of Questions

According to a Delphi prioritization procedure, the following three questions were selected: 1) Whether RA patients without fertility desire may achieve beneficial effects from the treatment of TGT/TWT alone at the active disease stage; 2) whether RA patients without fertility desire may achieve beneficial effects from the treatment of TGT/TWT in combination with Chinese patent medicine at the active disease stage; 3) whether RA patients without fertility desire may achieve beneficial effects from the treatment of TGT/TWT in combination with Western medicine at the active disease stage.

### Literature Review

Our literatures were collected from the following databases: 1) Chinese articles: China National Knowledge Infrastructure (CNKI), WanFang Data Resource, Chongqing VIP Information Database, and China BioMedical Literature Service System (SinoMed); 2) English articles: MEDLINE, Embase, and Cochrane Library. Articles published in Chinese or in English before January 2019 were selected. Literature retrieval was run using the keywords related to RA/TGT/TWT. The enrolled articles included the existing guidelines, systematic reviews, meta-analyses, RCTs, nonrandomized controlled trials, and observational studies.

### Level of the Evidence and Strength of the Recommendations

The level of the evidence was evaluated according to the guideline of GRADE Working Group (Balshem et al., 2011) (Table 1). The evidence-based recommendations were formed based on the strength of recommendations (Andrews et al., 2013) suggested by GRADE Working Group (Table 2). The expert-approved GPPs were formed through two rounds of Delphi survey and the consensus development conference (Liao and Xie, 2008).

**TABLE 1:** Significance of the four levels of evidence.

Quality level	Definition
High/I	We are very confident that the true effect lies close to that of the estimate of the effect
Moderate/II	We are moderately confident in the effect estimate: The true effect is likely to be close to the estimate of the effect, but there is a possibility that it is substantially different
Low/III	Our confidence in the effect estimate is limited: The true effect may be substantially different from the estimate of the effect
Very low/IV	We have very little confidence in the effect estimate: The true effect is likely to be substantially different from the estimate of effect

**TABLE 2:** Significance of the four strengths of recommendations.

Strength of the recommendation	Meaning for clinicians or patients
Strong/A	The panel is highly confident of the balance between desirable and undesirable consequences; they make a strong recommendation for (desirable outweighs undesirable) or against (undesirable outweighs desirable) an intervention
Weak/B	The panel is less confident of the balance between desirable and undesirable consequences; they offer a weak recommendation

### Development of Recommendations and GPPs

Eight recommendations and seven GPPs on the clinical application of TGT and TWT against RA were developed by taking real-name vote among the experts of the scientific committee and working group based on the following procedures. At first, the articles were carefully reviewed and the level of evidence offered by each article was given. Then, adverse effects, economic considerations, applicability of the recommendation to the target population of each recommendations, and GPPs were also shown in GRADE Evidence to Decision (EtD) framework. After that, GRADE EtD framework of the evidence-based recommendation was using the five-category scale (strongly disagree, disagree, uncertain, weak recommendation, and strong recommendation). The vote of any strongly disagree, disagree, weak recommendation, strong recommendation over 50% should be deemed to reach a consensus. The vote of any side of “uncertain” over 70% should be deemed to reach a consensus, and the strength of the recommendation should be recognized as weak. GRADE EtD framework of expert-approved GPP was using the three-category scale (consensus, uncertain, and against). The vote of any side of "uncertain" over 50% should be recognized as GPP.

### Peer Review

The guideline was assessed twice after the draft was completed by the guideline compiling team. For the first round of assessment, a total of 17 experts were invited to review the first draft of guideline. Following the revision, another 39 experts outside the working group were invited to review draft for the second time. After two rounds of experts’ assessment and amendment, the guideline was approved by the Standardization Office of the Chinese Association of Chinese Medicine.

### Publicity

The introduction and announcement of this guideline have been published on the official account of the Chinese Association of Chinese Medicine website. The guideline will be publicized in various academic conferences in the future.

### Update

The working group will update guideline within three years based on new evidence of TGT and TWT administrations against RA.

## Diagnostic Criteria

### Diagnosis in Western Medicine

1. The 1987 American College of Rheumatology (ACR) classification criteria for RA (Arnett et al., 1988).

Seven diagnostic criteria proposed by ACR are as follows: 1) morning stiffness in and around joints lasting at least 1 h before maximal improvement; 2) soft tissue swelling (arthritis) of three or more joint areas observed by a physician; 3) swelling (arthritis) of the proximal interphalangeal, metacarpophalangeal, or wrist joints; 4) symmetric swelling (arthritis); 5) rheumatoid nodules; 6) the presence of rheumatoid factor (RF); 7) radiographic erosions and/or periarticular osteopenia in hand and/or wrist joints. Criteria one through four must have been present for at least 6 weeks. RA is defined by the presence of four or more criteria, and no further qualifications (classic, definite, or probable) or list of exclusions is required.

(2) The 2010 ACR/European League Against Rheumatism (EULAR) classification criteria for RA (Aletaha et al., 2010).

The revised diagnostic criteria issued by ACR and EULAR in 2010 are as follows: 1) Target population (who should be tested?): patients 1) who have at least one joint with definite clinical synovitis (swelling); 2) with the synovitis not better explained by another disease. 2) Classification criteria for RA (score-based algorithm: add score of categories A-D; a score of 6/10 is needed for classification of a patient as having definite RA): A. Joint involvement: 1) large joint calculated as 0; 2) 2–10 large joints calculated as 1; 3) one to three small joints (with or without involvement of large joints) calculated as 2; 4) 4–10 small joints (with or without involvement of large joints) calculated as 3; 5) 10 joints (at least one small joint) calculated as five; B. Serology (at least one test result is needed for classification): 1) negative RF and negative Anti-Citrullinated Protein Antibody (ACPA) calculated as 0; 2) low-positive RF or low-positive ACPA calculated as 2; 3) high-positive RF or high-positive ACPA calculated as three; C. Acute-phase reactants (at least one test result is needed for classification): 1) normal C-reactive protein (CRP) and normal erythrocyte sedimentation rat (ESR) calculated as 0; 2) abnormal CRP or abnormal ESR calculated as one; D. Duration of symptoms: 1) <6 weeks calculated as 0; 2) ≥6 weeks calculated as 1.

### Disease Activity Classification

According to Disease Activity Score with 28-joint counts (DAS28) (Anderson et al., 2012), the disease activity of RA could be classified into four stages: clinical remission is defined by DAS28 < 2.6; low/minimal stage of RA is defined by 2.6 ≤ DAS28 < 3.2; moderate stage of RA is defined by 3.2 ≤ DAS28 ≤ 5.1; high/severe stage of RA is defined by DAS28 > 5.1; active stage of RA is defined by DAS28 ≥ 2.6; reaching the target to RA treatment is defined by DAS28 < 2.6.

### Diagnosis in Chinese Medicine (CM)

Those with pain and swelling of the small joints and morning stiffness as the chief complaint can be diagnosed with Wang Bi (National Administration of Traditional Chinese Medicine): 1) symmetric pain of the small joints, morning stiffness, and limited mobility are the initial performance of RA; 2) major characteristics of RA are as follows: slow onset, persistent, recurrent, and gradually angular wait; 3) with the progression of the disease, swollen joint, tenderness, and pain that worsens with activity appear gradually and eventually lead to stiffness or deformity of joints, muscular dystrophy, and rheumatoid nodules; 4) obvious changes into the presence of RF, ESR, radiographic erosions, periarticular osteopenia, subluxation and luxation of joint, joint ankylosis, and joint fusion at the active stage of RA appear.

## Actionable Recommendation

The aim of the administration of both TGT and TWT is to alleviate the disease severity of RA and inflammatory reactions, as well as to slow the progression of erosive osteoclasia.

### Suitable Crowd (GPP)

Both TGT and TWT are suitable for RA patients without fertility desire.

### Indication (GPP)

Both TGT and TWT are suitable for RA patients at the active stage (DAS28 ≥ 2.6).

### Directions (GPP)

TGT: For oral administration, the regular dose is 1 mg for every kilogram of the body weight, and the highest dose is restricted to 1.5 mg for every kilogram of the body weight, three times per day after meals.

TWT: For oral administration, the regular dose is 1–2 pills each time, three times per day after meals.

### Course of the Treatment (GPP)

Routine course of the administration of both TGT and TWT is three months. After that, DAS28 should be evaluated to confirm the therapeutic effects of the drugs. If DAS28 < 2.6, the dosage of both TGT and TWT should be reduced gradually.

### Combination Therapy (GPP)

If DAS28 is still more than 2.6 at the third month following the administrations of TGT or TWT, the combination therapeutics of TGT/TWT and methotrexate (MTX)/leflunomide (LEF)/total glucosides of white paeony capsules/Zhengqing Fengtongning tablet should be used for the treatment of RA.

### Both TGT and TWT are suitable for RA patients with all kinds of TCM syndromes (GPP)

Both “Chinese guideline for the diagnosis and treatment of rheumatoid arthritis (2018 version)” (Chinese Rheumatology Association) and “Guidelines for diagnosis and treatment of rheumatoid arthritis based on the combination of disease and syndrome” (Jiang et al., 2018) recommend TGT and TWT as effective Chinese patent medicine against RA. According to “Guidelines for diagnosis and treatment of rheumatoid arthritis based on the combination of disease and syndrome”, both TGT and TWT are suitable for RA patients with all kinds of TCM syndromes.

### The single administration of TGT against RA may alleviate the inflammatory reactions (GRADE A, Level III)

A meta-analysis showed that there were four randomized controlled trials (RCTs) that demonstrated ESR and CRP to be the prognostic markers for TGT administration against RA. TGT can significantly decrease CRP and ESR in RA patients, which is similar to MTX, MD_ESR_ = –2.66, 95%CI [–8.17, 2.86], *p* = 0.35; MD_CRP_ = –2.38, 95%CI [–9.01, 4.24], *p* = 0.48 (Li et al., 2019).

### The combined administration of TGT and MTX against RA may alleviate the inflammatory reactions (GRADE A, Level II)

A meta-analysis showed that there were 24 RCTs that demonstrated ESR, CRP, and RF to be the prognostic markers for the combined administration of TGT and MTX against RA. The combination of TGT with MTX can be more effective in reducing the levels of CRP, ESR, and RF than MTX alone, MD_ESR_ = 8.74, 95%CI [6.72, 10.76], *p* < 0.000 01; MD_CRP_ = 5.37, 95%CI [3.71, 7.03], *p* < 0.000 01; SMD_RF_ = 1.05, 95%CI [0.51, 1.60], *p* = 0.000 1 (Li et al., 2019).

### The single administration of TGT against RA may alleviate the symptoms (GRADE A, Level III)

A meta-analysis showed that there were three RCTs that demonstrated swollen joint count (SJC) and tender joint count (TJC) to be the primary outcome of the administration of TGT against RA. The therapeutic effects of TGT on alleviating joint swelling and tenderness in RA patients were similar to MTX, MD_SJC_ = 0.18, 95%CI [-1.06, 1.42], *p* = 0.78; MD_TJC_ = -0.06, 95%CI [-1.69, 1.56], *p* = 0.94 (Wang, X. Y et al., 2019).

### The combined administration of TGT and MTX against RA may alleviate the symptoms (GRADE A, SJC and TJC: Level II; MS: Level III)

A meta-analysis showed that there were 18 RCTs that demonstrated SJC, TJC, and morning stiffness time (MST) to be the prognostic markers of the combined administration of TGT and MTX against RA. The combination of TGT with MTX can more effective in reducing the levels of SJC, TJC, and MST than MTX alone, MDSJC = 3.01, 95%CI [2.09, 3.39], *p* < 0.000 01); MDTJC = 2.65, 95%CI [1.85, 3.44], *p* < 0.000 01; MDMST = 18.24, 95%CI [12.64, 23.84], *p* < 0.000 01 (Wang, X. Y et al., 2019).

### The single administration of TGT against RA may achieve good clinical efficacy (GRADE B, Level III)

A meta-analysis showed that there were three RCTs that demonstrated ACR criteria to be the prognostic criteria of the administration of TGT against RA. The clinical efficacy of TGT administration against RA patients was superior to MTX, RRACR = 1.31, 95%CI [1.15, 1.49], *p* < 0.000 1 (Chen et al., 2020).

### The combined administration of TGT and MTX against RA may achieve good clinical efficacy (GRADE A, Level III)

A meta-analysis showed that there were 10 RCTs that demonstrated ACR criteria to be the prognostic criteria of the combined administration of TGT and MTX against RA. The clinical efficacy of the combination of TGT with MTX was superior to MTX, RRACR = 1.28, 95%CI [1.20, 1.38], *p* < 0.000 1 (Chen et al., 2020).

### The combined administration of TGT and LEF against RA may alleviate the inflammatory reaction (GRADE A, Level II)

There were five RCTs that demonstrated CRP to be the prognostic markers of the combined administration of TGT and LEF against RA. Meta-analysis results showed that the combined administration of TGT and LEF was more effective in decreasing CRP than LEF alone, SMDCRP = 1.05, 95%CI [0.59, 1.52], *p* < 0.000 1 (Zhang et al., 2011; Min et al., 2013; Long and Li, 2014; Cui and Yang, 2016; Sun et al., 2016).

### The single administration of TGT against RA may slow the progression of erosive osteoclasia (GRADE B, Level III)

A meta-analysis showed that there were three RCTs that demonstrated the modified Sharp score (including joint erosions, JE; and joint space narrowing, JSN) to be the prognostic marker of the administration of TGT against RA. The therapeutic effects of TGT on slowing the progression of erosive osteoclasia in RA patients were superior to MTX and salazopyridine, RRJE = -1.37, 95%CI [-3.25, 0.51], *p* < 0.15; RRJSN = -1.25, 95%CI [-2.68, 0.18], *p* < 0.15 (Zhu et al., 2019).

### Toxic side effects should be monitored during the administration of both TGT and TWT (GPP)

Both the administrations of TGT and TWT may achieve good therapeutic effects in RA patients at the active stage. Because the bioactive constituents of *Tw*HF may often have toxicity, the adverse drug reactions (ADRs) occur occasionally. A meta-analysis (Li et al., 2020) showed that there were 79 studies that reported ADRs of the TGT administration against RA. Of 1,997 cases with ADRs in the experimental group, 792 (39.66%) were reported when TGT was used as the intervention. Of 1,361 cases with ADRs in the control group, 507 (37.25%) were reported when TGT was used as the intervention. The overall incidence of ADRs was 38.68% (1,299/3,358). Meta-analysis results of 49 studies with TGT as intervention in the experimental group showed that the overall incidence of ADRs was 0.23, 95%CI [0.22, 0.24], *p* = 0. ADRs mainly result in the damage of the reproductive, gastrointestinal, skin and accessories, blood, and hepatobiliary systems.

## Conclusion

Collectively, the development of clinical practice guideline for TGT/TWT in the treatment of RA may offer a clinical decision support for healthcare professionals according to the available evidence and expert opinions, which enables optimal patient care. Furthermore, this will also identify limitations in existing research and knowledge in *Tw*HF-based therapy that will form a research agenda for future investigations.

## Author Contributions

All authors listed have made a substantial, direct, and intellectual contribution to the work and approved it for publication.

## Funding

This study was supported by National Natural Science Foundation of China (81974526, 81974529, 81974537, and 81873068), Beijing Municipal Natural Science Foundation (7192139), and Fundamental Research Funds for the central public welfare research institutes (Z2017082 and ZXKT19013).

## Conflict of Interest

The authors declare that the research was conducted in the absence of any commercial or financial relationships that could be construed as a potential conflict of interest.
